# Mucoadhesive Gelatin Buccal Films with Propranolol Hydrochloride: Evaluation of Mechanical, Mucoadhesive, and Biopharmaceutical Properties

**DOI:** 10.3390/pharmaceutics13020273

**Published:** 2021-02-18

**Authors:** Marija Jovanović, Nataša Tomić, Sandra Cvijić, Dušica Stojanović, Svetlana Ibrić, Petar Uskoković

**Affiliations:** 1 Department of Materials Science and Engineering, Faculty of Technology and Metallurgy, University of Belgrade, Karnegijeva 4, 11120 Belgrade, Serbia; duca@tmf.bg.ac.rs (D.S.); puskokovic@tmf.bg.ac.rs (P.U.); 2 Innovation Center of Faculty of Technology and Metallurgy, Karnegijeva 4, 11120 Belgrade, Serbia; ntomic@tmf.bg.ac.rs; 3 Department of Pharmaceutical Technology and Cosmetology, Faculty of Pharmacy, University of Belgrade, Vojvode Stepe 450, 11221 Belgrade, Serbia; gsandra@pharmacy.bg.ac.rs (S.C.); svetlana.ibric@pharmacy.bg.ac.rs (S.I.)

**Keywords:** mucoadhesion, buccal films, gelatin, propranolol hydrochloride, in silico simulation

## Abstract

This study processes and characterizes propranolol hydrochloride/gelatin mucoadhesive buccal films. Two types of gelatin are used: Gelatin from porcine skin, type A (GA), and gelatin from bovine skin (GB). The influence of gelatin type on mechanical, mucoadhesive, and biopharmaceutical characteristics of buccal films is evaluated. Fourier-Transfer infrared spectroscopy (FTIR) and differential scanning calorimetry (DSC) analysis show that GA with propranolol hydrochloride (PRH) in the film (GAP) formed a physical mixture, whereas GB with PRH (GBP) form a compound-complex. Results of mechanical testing (tensile test, hardness) revealed that GAP films exhibit higher elastic modulus, tensile strength, and hardness. A mucoahesion test shows that GBP has higher adhesion strength, while GAP shows higher work of adhesion. Both in vitro release study and in silico simulation indicated that processed films can provide effective drug transport through the buccal mucosa. In silico simulation shows improved bioavailability from buccal films, in comparison to the immediate-release tablets—indicating that the therapeutic drug dose can be markedly reduced.

## 1. Introduction

Buccal mucosa is one of the promising drug administration sites that is becoming attractive for both local and systemic drug delivery [1,2]. Buccal mucosa is relatively permeable and has good vascularization [3,4]. This route offers many advantages compared to the oral route, such as avoiding gastrointestinal irritation and drug degradation and first-pass liver metabolism, which means it ensures better drug bioavailability [2,5,6]. Moreover, buccal mucosa is accessible, so that a dosage form can be easily administered, leading to better patient compliance compared to other drug dosing routes [5,6]. Buccal administration is particularly suitable for the pediatric and geriatric population, as well as patients that have problems with swallowing [6]. There are many different dosage forms for buccal administration, such as tablets, films, gels, patches, sprays, pastes [7]. Among them, mucoadhesive buccal films offer several advantages, due to the high flexibility and larger surface area for drug absorption. They ensure more accurate drug dosing compared to gels, which can be easily washed away by the saliva [2,7,8]. Also, in comparison to conventional buccal tablets, films can be more comfortable because they are thin, flexible, and also have good mechanical properties with a resistance to breakage caused by mouth movements [9,10]. Mucoadhesive films should have good tensile strength, mucoadhesive properties, and compatibility with the active substance [11].

Mucoadhesive natural polymers have received significant attention as carriers for buccal films, due to their ability to make close and prolonged contact with mucosa and to optimize drug bioavailability [10,12]. In this study, gelatin, the natural bioadhesive polymer, was selected to develop buccal mucoadhesive films. Gelatin is already widely used in pharmaceutical and medical applications and has been recognized as a GRAS (Generally Regarded as Safe) material by the United States Food and Drug Administration [13]. Gelatin has been used in the pharmaceutical field because of its excellent biodegradability, biocompatibility, non-toxicity, non-immunogenicity, affordability, etc. Gelatin, a natural protein derived from partially denatured collagen, is readily soluble in hot water and forms physically crosslinked hydrogels that are stable below its gelation temperature (≈23 °C) [14,15]. Polyelectrolyte complexes readily form between polyanions and polycations. These complex compounds are formed by the ionic association of repeating units on the polymer chains [16]. The stability of complex compounds is dependent on many environmental factors, such as a solvent’s nature, pH, and ionic strength [17,18]. There are two types of gelatin: Type A gelatin (GA), derived from acid-treated processes; and type B gelatin (GB), derived from alkali-treated processes [19]. Different production processes affect the isoelectric point (pI), pH, and other properties of GA and GB. GA has a pI between 8 and 9 (positive charge at neutral pH), while GB has a pI between 4.8 and 5.4 (negative charge at neutral pH) [20].

Mucoadhesive strength points to the feasibility of films in terms of their adherence to mucosal membrane. Excessive adhesion may cause harm (irritation) to mucosa, whereas inadequate adhesion will adversely affect therapeutic efficacy, and at the same time, it may cause patient non-compliance. Hence, an optimized mucoadhesion is required for a film to be effectively used as a drug delivery system [21]. There are four possible general interactions between mucoadhesive polymers and glycoproteins: (1) Covalent attachment; (2) electrostatic interaction, which requires matching of charge groups between the polymer and the mucus; (3) hydrogen bonding; and (4) hydrophobic interactions [22].

Propranolol hydrochloride (PRH) is a non-selective β-adrenergic blocking agent (β-blocker) and has been used in the treatment of hypertension, arrhythmias, angina pectoris, and many other cardiovascular diseases; moreover, it can be used for migraine prophylaxis, tremor, and anxiety treatment. PRH has low bioavailability after oral administration, due to the extensive first-pass metabolism. This can be a problem because it is reported that approximately only 25% of PRH reaches systemic circulation [2,23]. PRH shows pH-dependent solubility; solubility at pH 1.2 is 225 mg/mL, while at pH 6.8 it is 130 mg/mL [24,25]. In addition, the drug is stable when the pH level is acidic and decomposes rapidly when it is alkaline. Solutions are most stable at pH 3; in aqueous solutions, propranolol decomposes, due to the oxidation of the isopropylamine side-chain [25].

In this study, PRH has been chosen as a model drug because it has suitable physicochemical properties (MW 259.34 g/mol, logP = 3.22), it is a BCS class 1 drug (it has high solubility and permeability), and since it undergoes the first-pass metabolism, patients may benefit from buccal administration [10]. There are many papers that describe the research of PRH in different buccal formulations. Several polymer blends were used. Arbuzzo et al. investigated matrices of chitosan and gelatin for buccal delivery of propranolol hydrochloride. Their group made PRH buccal films [4] with different ratios of these polymers; buccal tablets with chitosan/gelatin microparticles [26]; bilayered buccal films based on polyvinylpyrrolidone or polyvinylalcohol with different weight ratios of gelatin or chitosan [10]. Kraisit et al. developed mucoadhesive buccal film based on hydroxypropyl methylcellulose and polycarbophil loaded with PRH nanoparticles [27]. Patel et al. prepared buccal films with different ratios of chitosan and polyvinylpyrrolidone K-30 [28]. Salehi and Boddohi developed mucoadhesive buccal film for co-delivery of rizatriptan benzoate and propranolol hydrochloride using kollicoat^®^ IR, polyethylene oxide, and hydroxypropyl methylcellulose [2].

Based on our best knowledge, the comparison of two types of gelatin A and gelatin B as drug carriers for buccal film has not been performed. Aramwit et al. investigated gelatin A and gelatin B nanoparticles as carriers for controlled drug release [19].

This study aims to comprehensively characterize propranolol hydrochloride/gelatin buccal mucoadhesive films prepared with two types of gelatin: Gelatin from porcine skin, type A (GA), and gelatin from bovine skin (GB). The study was focused on possible drug/carrier interactions and their impact on the mechanical, mucoadhesive, and biopharmaceutical characteristics of the film. Additionally, the bioavailability of the obtained mucoadhesive films was compared with commercially available PRH IR tablets using in silico simulation in GastroPlus™ software. The results from this study will allow researchers the choice of suitable gelatin type to obtain the desired drug release profile.

## 2. Materials and Methods

### 2.1. Materials

Propranolol hydrochloride (PRH) (Galenika a.d., Belgrade, Serbia), which was used as a model substance, was Ph. Eur. 10 grade. Type A gelatin from porcine skin (~300 g Bloom) and type B gelatin from bovine skin (~225 g Bloom) were obtained commercially from Sigma–Aldrich (Sigma–Aldrich Co., St. Louis, MO, USA). Glacial hydrochloric acid (M = 60.05 g/mol), which was used as a solvent, was obtained from Zorka Pharma (Zorka Pharma HEMIJA d.o.o., Sabac, Serbia). Deionized water (DI) (resistance of 18 MΩ cm) was used to prepare the solutions. For the mucoadhesion studies, the mucin from a porcine stomach, Type II (Sigma-Aldrich Co., St. Louis, MO, USA), was used. Potassium hydrogen phosphate, potassium dihydrogen phosphate, sodium chloride, and purified water were used to prepare simulated saliva (pH 6.8 was adjusted with 0.1 M hydrochloric acid) [4].

### 2.2. Methods

#### 2.2.1. Preparation of Gelatin Films

**Figure 1:**
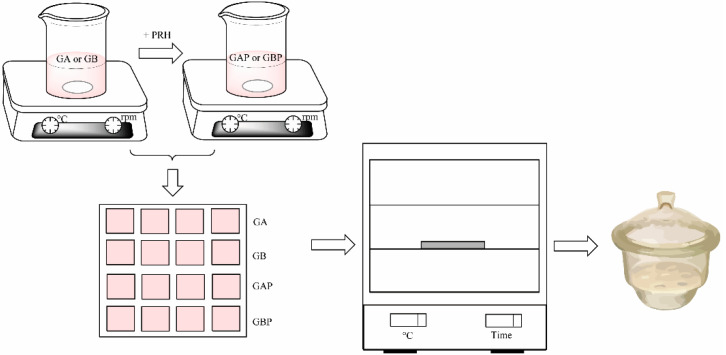
Scheme of the film preparation.

**Figure 2:**
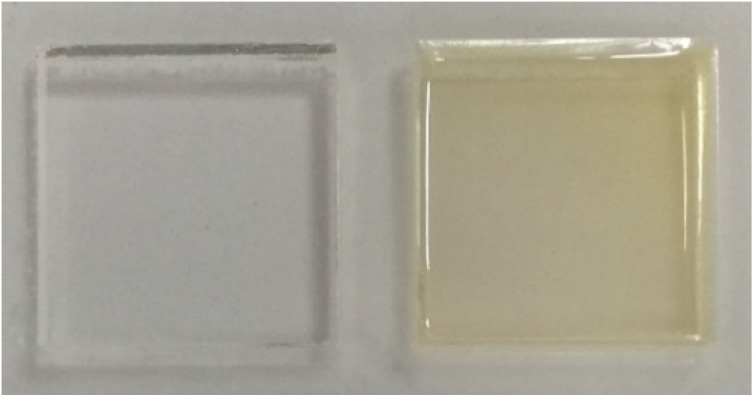
Mold (**left**) and the sample of the film (**right**).

Four series of films were prepared: Pure gelatin A (GA), gelatin A with propranolol hydrochloride (GAP), pure gelatin B (GB), and gelatin B with propranolol hydrochloride (GBP). The films were produced by solvent casting method, as shown in Figure 1. Gelatin A or gelatin B was added to the water solution of the glacial acetic acid (20% *w/w*) to create a 20% polymer solution. It was then stirred on a magnetic stirrer for 3 h at room temperature. The procedure for PRH loaded films was the same: After obtaining 20% polymer solution, the specific amount of propranolol hydrochloride was added and stirred for 3 h at room temperature to obtain a homogeneous solution. After that, it was poured into a mold (Figure 2) and dried at room temperature for 72 h for the solvent to evaporate. After that, it was dried in a hot air oven at 30 °C for 6 h to evaporate any residual solvent. After the films were dried, they were removed from the mold, cut in an appropriate sample size (15 mm × 15 mm × 0.6 mm), packed in aluminum foil, and stored in a desiccator (NaBr saline saturated solution, relative humidity = 58%) at 25 °C for at least 48 h until analysis [29]. The amount of gelatin in the film was determined as follows: The films (dimensions 15 mm × 15 mm × 0.6 mm) were weighed, then dried in a hot air oven at 100 °C to constant weight. After drying, the films were weighed, and the amount of gelatin was calculated by subtracting the drug content from the mass of the film. For each formulation, assays were performed in triplicate. The composition of the sample film was 30 mg of PRH and 130 mg of gelatin A or B.

#### 2.2.2. Fourier-Transform Infrared Spectroscopy (FT-IR)

Single-beam Fourier-Transfer infrared spectroscopy (FTIR) using a Nicolet 6700 spectrometer (Thermo Fisher Scientific, Waltham, MA, USA) in the attenuated total reflectance (ATR) mode using a single bounce 45 °F Golden Gate ATR accessory with a diamond crystal, and an electronically cooled DTGS detector. The spectra were the co-addition of 64 scans at 4 cm^−1^ spectral resolution, and were ATR corrected. The Nicolet 6700 FT-IR spectrometer was equipped with OMNIC software (Thermo Fisher Scientific, Waltham, MA, USA) and recorded the spectra in the wavelength range from 2.5 μm to 20 μm (i.e., 4000 cm^−1^ to 500 cm^−1^).

#### 2.2.3. Differential Scanning Calorimetry (DSC)

The thermal analysis of films was performed on a device for differential scanning calorimetry (DSC) in a temperature range from 25 °C to 185 °C (Q10, TA Instruments, Crawley, UK) under a dynamic nitrogen flow of 50 mL/min. Samples of 7–9 mg were investigated. The samples were heated up at a rate of 10 °C/min. The glass transition temperature (T_g_) was determined at the midpoint of the step-transition for each sample. The T_g_ values were confirmed by the use of the derivative curve [30].

#### 2.2.4. Field Emission Scanning Electron Microscope (FESEM)

An observation of film morphology was performed using FESEM TESCAN MIRA 3 (Tescan Orsay Holding, a.s., Brno – Kohoutovice, Czech Republic), with fractured surfaces of the films sputtered with gold.

#### 2.2.5. Mechanical Characterization

##### Tensile Strength

The uniaxial tensile mechanical test was performed using Shimadzu Servopulser (Shimadzu Corporation, Kyoto, Japan) according to ASTM D638 [31]. Measurements were taken with a cross-head speed of 2 mm/min at 25 °C. The samples were in the shape of film strips 50 × 10 × 0.6 mm. The distance between the jaws was 22 mm. Young’s modulus (modulus of elasticity) was calculated from the slope of the linear part of the tensile curve [32]. Three measurements were made for each sample and tested. The average values of the tensile strength, modulus of elasticity, and elongation at break were determined.

##### Hardness Measurement (Vickers)

The hardness test was performed to investigate the mechanical response of films to indentation. Vickers method consists of indenting the test material with a diamond indenter, in the form of a right pyramid with a square base and an angle of 136 °. The indent was in the shape of a pyramid sheath resembling a square, and the diagonal length was then measured. The micro-hardness of the samples was also measured, using Vickers micro-hardness tester DURIMET I Kleinharteprufer (Leitz, Wetzlar, Germany) with a load of 4.9 N according to ASTM E384-16 [33]. Three indentations were performed, obtaining six diagonal lengths for the calculation of hardness.

#### 2.2.6. Determination of Mucoadhesive Properties of Films

Mucoadhesion studies of formulated films were performed on Texture Analyzer Shimadzu EZ Test LX (Shimadzu Corporation, Kyoto, Japan). Mucin disks were used as a mucosal substrate. They were made from 250 mg of mucin powder that was compressed into a 13 mm diameter disk [34]. The simulated saliva was heated and maintained at 37 °C. A mucin disk was placed in the center hole of the acrylate holder, and attached with a double adhesive tape. The film (circle, 10 mm diameter) was fixed onto the metal rod of the texture analyzer. Mucin disk was submerged in 3 mL of the simulated saliva for 120 s, and then the excess was drawn-out with a syringe [8,35]. The holder with soaked mucin disk was being placed bellow metal rod of texture analyzer on which our sample was attached. Experimental conditions were: Sixty seconds of contact time of mucin disk and our sample with the force of 1 N, and then the movable metal rod was being lifted at a speed of 0.1 mm/s until the sample was totally separated from the mucin disk. The used program Trapezium X (Shimadzu Corporation, Kyoto, Japan) automatically determines the force of adhesion (maximum force required to detach sample from a mucin disk) and work of adhesion (the area under the force/time curve) [34].

The data obtained through experimental results were analyzed by using a two-way analysis of variance (ANOVA, OriginLab Corporation, Northampton, MA, USA) at a confidence level of *p* < 0.05. Polymer type and drug presence were used as factors in the analysis as independent variables, and force and work were used as dependent variables. The statistical analysis was performed in the Origin software.

#### 2.2.7. Drug Content Uniformity

The casted films were cut from three different places to evaluate drug content uniformity [2]. Then drug content measurement was carried out by completely dissolving each sample in 250 mL of distilled water (pH = 6.8). After filtration of solutions through 0.45 μm filter paper, the absorbance of PRH was measured at 319 nm. The calibration curve was used for the determination of PRH concentrations in each film. The following equation was used for the calculation of drug content uniformity, and the average value was reported. The limit of uniformity of drug content should be in the range of 85 to 115% [2,11].

Drug’s content uniformity = (Actual amount of drug in film * 100)/Theoretical amount of drug in the film)

#### 2.2.8. In Vitro Drug Release Studies

**Figure 3:**
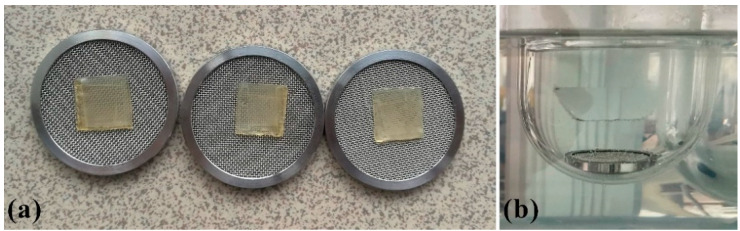
(**a**) Samples on metal mesh disc; (**b**) set up for dissolution test.

Rotating paddle over disk apparatus Erweka DT70 (Erweka Gmbh, Langen, Germany) was used, at the rotating speed of 50 rpm. 200 mL of deionized water (pH 6.8) served as a dissolution medium, at 37 ± 0.5 °C [8,28,36,37,38,39,40,41]. A single film (15 × 15 × 0.6 mm) was placed on a metal mesh disc (Figure 3) fixed with the help of a two-sided adhesive tape to avoid floating of the film during testing. The film was put on the upper side of the disc, together with the disc, immersed in the dissolution vessel [28,42,43]. Samples of 4 mL were taken from the medium at fixed time intervals (5, 10, 15, 30, 45, 60 min) and replaced with a fresh dissolution medium [44]. All samples were filtered through a 0.45 µm membrane filter (Millipore, Bedford, MA, USA). The concentration of PRH was determined by UV/VIS spectrophotometer Evolution 300 (Thermo Fisher Scientific, Cambridge, UK) at 319 nm. The results were shown as the medium value of three repeated measurements for each sample [45].

#### 2.2.9. Physiologically-Based Simulations

Propranolol absorption and the concomitant disposition were simulated using GastroPlus™ software (v. 9.7.0009, Simulations Plus Inc., Lancaster, CA, USA). The software accounts for the three types of input data: Drug physicochemical and pharmacokinetic properties, along with physiological data stored with the software integrated Advanced Compartmental Absorption the Transit (ACAT) model and the Oral Cavity Compartmental Absorption and Transit (OCCAT) model. Both models use a system of differential equation to simulate drug transport and absorption, whereas the ACAT model describes drug performance in the gastrointestinal tract (nine consecutive segments from the stomach to the ascending colon), and the OCCAT model depicts drug transit and absorption through the oral cavity mucosa (conforming of six compartments: Buccal, gingival, palate, the top and the bottom of the tongue, the floor of the mouth). Details about the ACAT and OCCAT models are described in the literature [46,47]. We have used our previously generated and validated propranolol-specific model [48]. The selected drug-related input parameters were literature values, experimentally determined, or in silico predicted data. The selection of input data is shown in Table 1. The ACAT model parameters were software default values for human adult fasted physiology. The OCCAT model physiological parameters were also default values, except for the saliva production rate, which was optimized at 0.6 mL/min, as explained in our previous paper [48]. The increased saliva production rate in comparison to default 0.04 mL/min can be justified by the presence of a “foreign body” in the oral cavity [38]. Namely, published data demonstrate that saliva production rate can be increased up to 2.34 mL/min [49] or even 6.17 g/min [50], depending on the type of stimulus and the exposure time, justifying the increase in saliva production rate value considered in our model. Formulation-specific input data describing the designed formulations GAP and GBP included the drug dose (30 mg), formulation type (“buccal patch”), and experimentally determined drug release profiles. We need to note that “buccal patch” is currently the only software option that allows the input of in vitro dissolution data for dosage forms absorbed from the oral cavity, and therefore, it was selected to estimate the in vivo performance of buccal films. However, the assumption that the contact surface area of the film with buccal mucosa is constant may be somewhat erroneous.

## 3. Results and Discussion

### 3.1. FTIR Analyses

**Figure 4:**
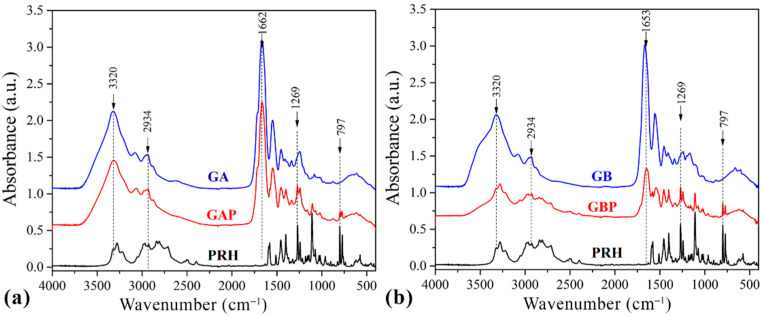
Fourier-Transfer infrared spectroscopy (FTIR) spectrums of (**a**) pure gelatin A film (GA), propranolol hydrochloride (PRH), and gelatin A film loaded with PRH (GAP); (**b**) pure gelatin B film (GB), propranolol hydrochloride (PRH), and gelatin B film loaded with PRH (GBP).

The drug–polymer interaction was checked by comparing the FTIR spectra mixture of the drug containing the GA and GB, with the FTIR spectrum of the pure drug and the pure GA and GB (Figure 4.).

Pure GA showed the bands at 1662 cm^−1^ relative to the vibration of the amide carbonyl group; at 1543 cm^−1^ it was associated with stretching of the free amino groups [4,15,57]. The peak at 1243 cm^−1^ corresponds to CN stretching, NH in-plane bending, and CH_2_ wagging vibrations [58,59]. The broad absorption band at 3320 cm^−1^ is assigned to the OH stretching and internal hydrogen bonds, which overlap with N−H stretching [57].

The spectrum of GB showed the bands at 1653 cm^−1^ corresponding to the vibration of the amide carbonyl; at 1557 cm^−1^ it was associated with the stretching of the free amino groups [4,15], 1234 cm^−1^ was attributed to combination peaks between C−N stretching vibrations, N−H and the wagging vibrations from CH_2_ groups stem from close amino acid residues [60,61]. These peaks are known as Amides I, II, and III. 

The spectrum of PRH shows peaks at 2965 cm^−1^, due to the presence of a secondary amine group; 3283 cm^−1^ is due to the hydroxyl group (secondary), the C−O−C stretching in aryl alkyl ether display a stretching band at 1108 cm^−1^, and the peak at 797 cm^−1^, due to a-substituted naphthalene [2,24,62]. Frequencies of the functional groups of the pure drug remained intact in the mixture with GA (GAP); hence, there was no major interaction between the drug and the GA.

In this part, the electrolytic nature of gelatin and PRH could be considered. Their electrically charged ions could interact in solution in direction to form polyelectrolytic complex (PEC), and Coulomb forces are responsible for this behavior.

The FTIR spectrum of the complex in comparison with the physical mixture is shown in the spectrum for PRH with the GB (GBP). The characteristic carbonyl absorption in 1596 cm^−1^ is replaced by the band in the 1550–1560 cm^−1^ region. This band corresponds to the auto-symmetrical vibrations of the COO-structure and is used as a diagnosis for the COO-group [16,61]. This ionization leads to the possible formation of anion-cation interaction. According to the literature, the formation of PEC should be confirmed with FTIR, DSC, and drug release profile [4,8,16,56,63,64].

FTIR analysis revealed that in GAP, there is no interaction between PRH and GA, while for GBP some results indicate ion-ion reactions. This should be confirmed after DSC analyses and dissolution test.

### 3.2. DSC Analyses

**Figure 5:**
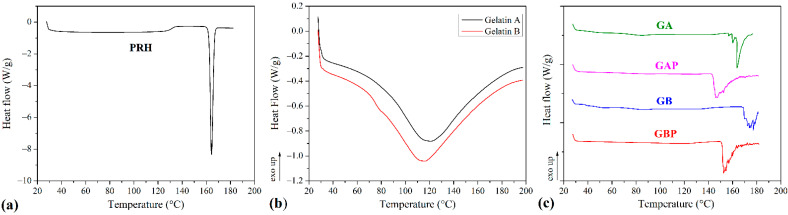
Differential scanning calorimetry (DSC) diagrams of (**a**) propranolol hydrochloride (PRH); (**b**) gelatin A and gelatin B powder, (**c**) pure gelatin A film (GA), gelatin A film loaded with PRH (GAP), pure gelatin B film (GB) and gelatin B film loaded with PRH (GBP).

The results of the DSC analysis of processed series and powders of Gelatin A and Gelatin B (as received) are presented in Figure 5 and Table 2. The curve of the pure PRH shows the melting point at 163 °C [4,10]. The curves for powders Gelatin A and Gelatin B show the points of transition temperatures at 30.41 °C and 32.35 °C, respectively. Also, there are endothermic peaks for Gelatin A and Gelatin B powders at 121.06 °C and 115.82 °C, respectively. For pure GA film, the points of transition temperature at 44.52 °C and endothermic peak at 85.75 °C were observed [65]. The glass-to rubber transition associated with polymer molecules started to move and vibrate, and viscoelastic behavior instead of the solid body ensued [66,67,68]. The endothermic peak is associated with the melting of the triple-helix crystalline structure. This peak is followed by processes, such as water evaporation, melting, and recrystallization, of small and/or imperfect gelatin crystallites. Also, because gelatin consists of peptides and proteins produced by partial hydrolysis of collagen, this peak could be obtained, due to overlapping of the glass transition of α-amino acid blocks in the polypeptide chain [68,69,70,71,72]. The appearance of this peak is depended on the film preparation conditions (drying). For GB film, those relevant temperatures associated with T_g_ and endothermic peak were 48.79 °C and 87.89 °C. With the addition of PRH, these temperatures were raised to 53.11 °C and 86.09 °C for GAP and for GBP 60.09 °C and 94.26 °C. There was also the third transition with peak temperature associated with the isomerization of the peptide bonds in gelatin from the trans to the cis configuration and marked as Ti [73,74,75]. For GA and GB films, the values of Ti were 163.33 °C and 177 °C, respectively. It is also found that Ti is correlated with the moisture of gelatin film, and raised when moisture decreased [75,76]. In thermograms for films with PRH, Ti was shifted to lower temperatures, 147.32 °C for GAP and 152.55 °C for GBP. The irregularity and shifting of peaks could be attributed to the presence of acetic acid [60].

According to FTIR analysis, GAP is a physical mixture, while GAB is a complex compound. The DSC results have not provided clear confirmation of the interaction between gelatin and PRH. The rising of characteristic temperatures could be due to the crosslinking of polymer chains with PRH. [16,68]. The higher shift of T_g_ for GAP, then for GBP (accompanying with FTIR analysis), could lead to distinguish the nature of the interaction between gelatin A or B and PRH.

In both samples with the drug (GAP and GBP), a PRH crystallization peak was not found. The amorphous form was therefore obtained, indicating better solubility and bioavailability for both films [10].

### 3.3. SEM Analysis

**Figure 6:**
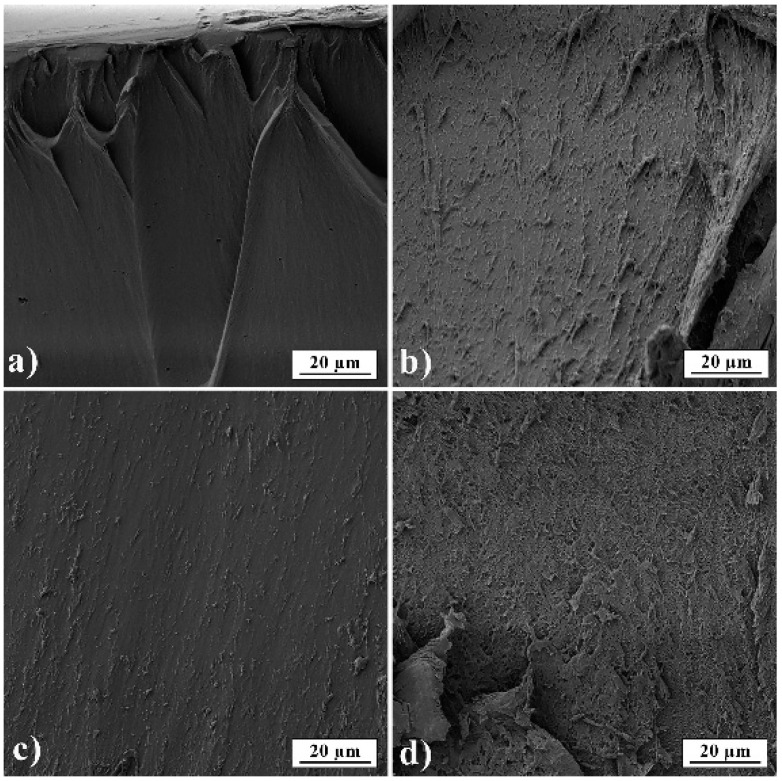
SEM micrographs of films’ fractured surfaces (**a**) GA; (**b**) GB; (**c**) GAP; (**d**) GBP.

In Figure 6, film cross-sections of pure gelatin A and gelatin B and films loaded with PRH are presented. The images show that films are smooth and nonporous. It can also be seen that films have good homogeneity and no presence of PRH powder.

### 3.4. Mechanical Properties

**Figure 7:**
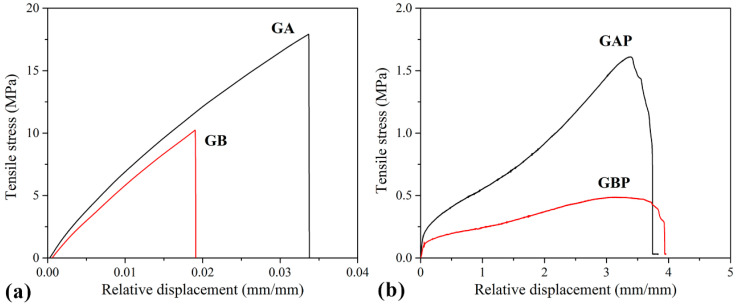
Tensile stress–strain curves of: (**a**) Pure gelatin films (GA and GB); (**b**) films with PRH (GAP and GBP).

Tensile curves and the results of the tensile test are presented in Figure 7 and Table 3. The mechanical properties of natural polymers are influenced by the preparation process, temperature, and moisture, so it is difficult to compare them with other references. However, it could be seen that the pure GA and GB results are in agreement with some of the data from the literature [32]. Film GA with a higher bloom index showed higher tensile strength, elastic modulus, and elongation at break, as expected [32]. For mucoadhesive buccal films, there are no recommended specific values for tensile strength and modulus of elasticity. Results obtained in this study for GAP and GBP are in range with the other published results for mucoadhesive buccal films with different compositions (i.e., hydroxypropyl methylcellulose (HPMC)/polycarbophil (PC) blend, polyvinylalcohol (PVA), and polyvinylpyrrolidone (PVP) blend and more complex composition [27,37,40,77,78,79,80].

It can be noted that the tensile strength and elastic modulus decreased with the addition of PRH both in GA and GB, while the elongation at break increased by three orders of magnitude. Also, it could be seen from the curves’ shapes that the plasticity of the films with PRH was raised. The addition of drug in gelatin leads to higher free volume and make the easier moving of polymer molecules. In pure gelatin, chains are tangled. During solvent casting, drug molecules were positioned between the polymer chains and increased the possibility of film deformation.

**Figure 8:**
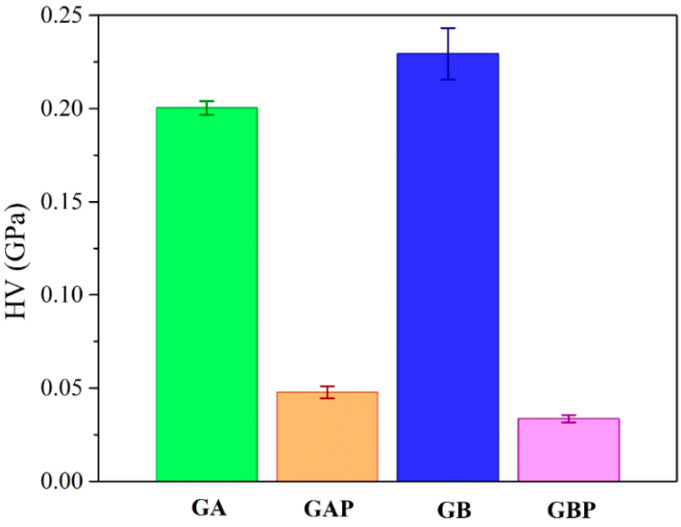
Results of Vickers micro-hardness test.

The results of micro-hardness are presented in Figure 8. Hardness represents the ability of materials to respond to plastic deformation. GA showed a slightly lower hardness than GB. Regardless of the physical mixture in GAP in comparison to the complex in GBP, the hardness of GAP was higher than for GBP. The addition of PRH in GA acted as physical crosslinking of the polymer, while in GBP, a complex was formed [16,19].

### 3.5. Mucoadhesion Studies

When two parts of material are connected together via surfaces, the force of their detachment is the measure of their adhesion [81].

A test for mucoadhesion was performed in accordance with the fracture theory, and the results are presented in Table 4. In fracture theory, the force required for the detachment of a film is related to the strength of the adhesive bond, while work of adhesion (W_adh_) is total energy involved for this separation, and it can be calculated as the area under force–distance curve [82]. It could be seen that the strength of adhesion (detachment force, F_adh_) of the gelatin increases with the addition of PRH. The force of adhesion for pure GB is higher than for pure GA for almost 10%. Strength of adhesion increased with the addition of PRH to the gelatin, both for GAP and GBP in relation to pure GA and GB. The energy or the work of adhesion (W_adh_) is associated with crosslinking of polymers; it is higher when the crosslinking is reduced. Polymers with lower crosslinking easily diffuse and entangle with mucin fibers. From Table 4, it can be observed that adding PRH to GA leads to higher energy. The bonding of mucin and film may be either primary (like covalent bonds) or secondary, like van der Waals bonding, hydrogen bonding, hydrophobic inter-actions, or electrostatic forces. This result should be combined with an electrostatic theory about electro attraction between anionic polymers (GA) with negatively charged sialic acid and sulfate residues of mucin glycoprotein [10,76,81,82]. For GB, adding PRH leads to a lower value of the work of adhesion. W_adh_ is the energy associated with surface deformation in the detachment and fracture mode of adhesive material. During detachment of two surfaces occur the crack formation and its propagation. The behavior of GBP in adhesion test shows viscoelasticity, while a brittle detachment fracture occurs. Like in the case of tension, when toughness is determined as the area under stress–strain curve, it is possible to maintain higher force with the lower area under the curve. The reversible work of adhesion could be described as the sum of the components corresponding to molecular interaction, surface deformation, and instabilities in adhesion and subsequent separation [83,84]. Moreover, it could be said that the lower W_adh_ implies a higher crosslinking of the polymer. This state causes a slower intake of fluid into the polymer and decreases the rate of polymer molecules and mucin interpenetration [22]. As the crosslinking density of the polymer is increased, the diffusion of fluid decreases. The slowdown in diffusion is more severe at the polymer-water interface [85].

The work of adhesion for GAP is the highest. The presence of a higher number of amino groups leads to a lower density of crosslinking. Due to this fact a much better intake of water into the film occurs, and all of this results in swelling. On the other hand, the highest force of adhesion occurs with GBP, but at the same time, the lowest work of adhesion can be observed. That means that, in this way, the highest density of crosslink is achieved.

Table 5 shows the results of the statistical analysis for force as the dependent variable and Table 6 for work as the dependent variable.

It can be concluded from the results of the statistical analysis that neither of the factors nor their interaction is statistically significant, as all p values are higher than 0.05.

### 3.6. Drug Content Uniformity

Drug content uniformity percentage is in the range of 98.61 ± 3.35 for GAP and 99.28 ± 3.74 for GBP. The results showed that there were acceptable changes in drug’s content, so the films have uniform drug distribution.

### 3.7. In-Vitro Release Study

The release of the drug from the buccal film is determined by drug/polymer properties. It also depends on the drug solubility, the drug diffusion from the film, the swelling, and degradation of the polymer matrix [42,57,86,87]. Although propranolol hydrochloride solubility is not expected to be the rate-limiting step for its dissolution and absorption [52], a dissolution study was performed under pH conditions that correspond to the physiological pH in the oral cavity (pH of the deionized water was 6.8). Other properties of the oral cavity fluid, such as ion concentration, osmolality, surface tension, viscosity, etc., have not been considered in this study; however, swelling and dissolution of some natural polymers may depend on these conditions. In this case, the diffusional force is much more important for the drug release than the films’ degradation because of the gelatin concentration of 20% [57,87]. Therefore, it is very important to evaluate in vitro release profile of the buccal film [42]. 

**Figure 9:**
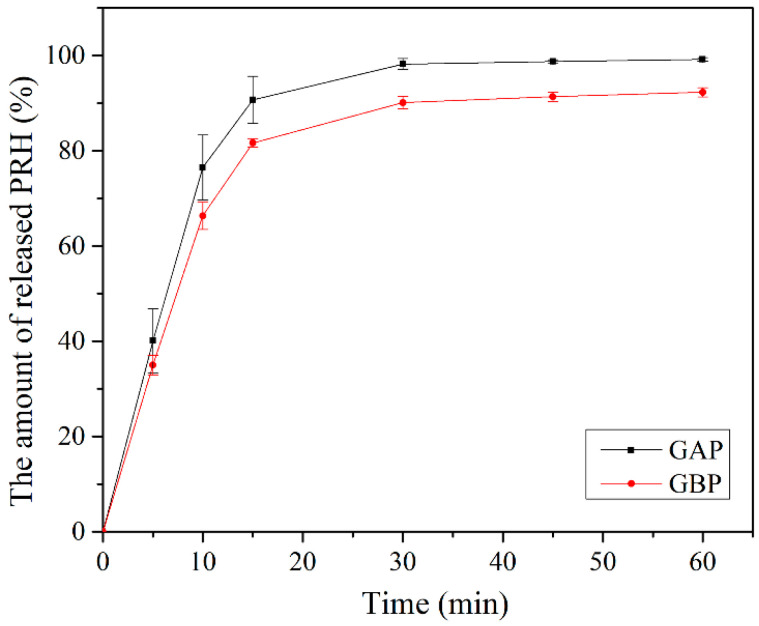
In vitro release study.

In vitro release of PRH from GAP and GBP, films are shown in Figure 9. Drug release happened immediately out of both formulations. More than 50% of PRH was released in the first 10 min and more than 80% in the first 15 min. The release was better from GAP films because the polymer and the drug are a physical mixture in comparison to GBP, where the complex was formed, which was shown on the FTIR spectra and the DSC analysis. There was a complete release of PRH from GAP, and a bit less from GBP because of the complex formation [4,10]. Moreover, the density of crosslink has a role in the release of PRH, as was previously mentioned in the mucoadhesion discussion.

Here, we also need to note that under the in vivo conditions, a drug can diffuse through both sides of the film, but since the designed formulations were intended for immediate drug release, as confirmed by our dissolution test results, the applied test conditions can be considered acceptable.

### 3.8. Physiologically-Based Simulations

Using the input data noted in Table 1, together with the drug dissolution profiles, shown in Figure 9, the expected propranolol absorption and disposition pattern has been predicted following the buccal application of the designed films. Here we adjusted the simulation setup (i.e., contact surface area) to comply with the dissolution test conditions, which is justified by the obtained fast drug release profiles, but as implied earlier, the drug diffusion can occur through both sides of the film under in vivo conditions.

**Figure 10:**
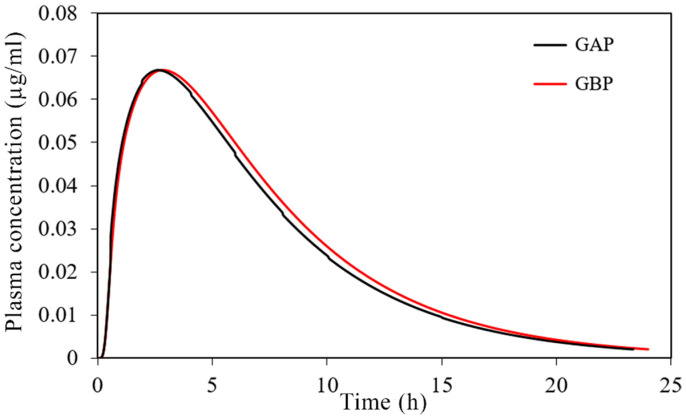
Predicted plasma concentration-time profiles for the tested 30 mg PRH buccal films.

The simulated plasma concentration-time profiles are depicted in Figure 10, and the corresponding pharmacokinetic parameters are given in Table 7.

**Figure 11:**
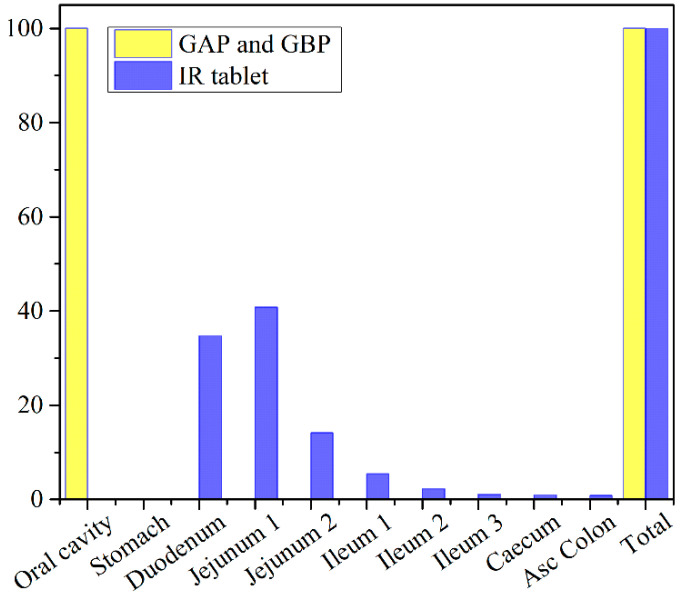
Predicted regional absorption profiles for the tested 30 mg PRH formulations and 80 mg PRH IR tablets.

The obtained results indicate negligible differences in the rate and extent of propranolol absorption from the tested formulations, with only about a 3% decrease in t_max_ and a 2% decrease in area under the plasma concentration-time curve (AUC_0–∞_) for the formulation GAP in comparison to GBP. These slight differences are caused by a somewhat slower drug dissolution rate from the formulation GBP, but in general, the profiles can be considered similar. The same congruency applies for the simulated regional drug absorption from the tested films, indicating complete absorption from the buccal region (Figure 11). Such high bioavailability can be considered a substantial benefit of propranolol buccal films [5,7,88,89,90]. Namely, in comparison to commercially available oral dosage forms, e.g., 80 mg propranolol IR tablets, the drug absorption is complete for both buccal and oral dosing routes (Figure 11 and Table 5), but due to the extensive first-pass metabolism in the liver, the drug bioavailability following oral dosing is notably decreased (Table 7). Due to the improved propranolol bioavailability from buccal films, the therapeutic drug dosing or dosages can be markedly reduced, e.g., the extent of drug absorption, expressed as AUC, obtained from 30 mg PRH buccal films is in the same range as obtained from 80 mg PRH IR tablet (which is less than 10% difference). Future in vivo studies are encouraged to support our in silico prediction results.

## 4. Conclusions

In this work, two types of gelatin (gelatin from porcine skin, type A (GA), and gelatin from bovine skin (GB)), were compared as suitable candidates for PRH carriers in the form of mucoadhesive buccal films. A comprehensive characterization was implemented to gain insight into the phenomenology of processing, morphology, mechanical and mucoadhesive behavior, and the drug delivery as well. PRH was chosen for the model drug because in a buccal film, it has a possibility to avoid loss during extensive first-pass metabolism. Elucidation of pure gelatin films structure (GA and GB) and also ones loaded with PRH (GAP and GBP) were investigated and compared.

Both types of drug-loaded films have shown good morphological homogeneity and have contained the amorphous form of PRH, which leads to better bioavailability of the drug. This was also confirmed by in silico simulation of regional absorption profiles, and due to the improved PRH bioavailability from buccal films in comparison with the immediate-release tablets, the therapeutic drug dose can be markedly reduced. The obtained GAP film shows 2.5 times higher elastic modulus, more than four times higher tensile strength, and 33% higher hardness in comparison to GBP film. Statistical analysis of the mucoadhesion tests have shown that neither polymer type nor drug nor their interaction had a statistically significant influence the strength and work of adhesion. Both films, GAP and GBP, have a similar drug release profile, with a slightly slower dissolution rate for GBP. Some tests and analyses were conducted regarding the ionic nature of GA, GB, and the drug. The dissolution test, FTIR, and DSC analysis together indicate that in GBP, the ion-ion complex interaction was more pronounced, while GA formed a physical mixture with PRH. The results presented in this work allowed us to estimate that GAP film will have better processing availability and higher solubility followed by faster drug release. On the other hand, GBP showed slightly lower mechanical strength and slower drug release profile, but stronger mucoadhesion force. In conclusion, gelatin-based mucoadhesive films with PRH have good potential for drug delivery to the buccal mucosa, allowing targeted delivery and reduced drug dosing.

## Figures and Tables

**Table 1:** Model input parameters.

Parameter	Value
Molecular weight	259.34 g/mol
logP	3.22 ^a^
pKa (base)	9.09 ^b^
Solubility	225 mg/mL (pH 1.2); 130 mg/mL (pH 6.8) ^c^
Diffusion coefficient	0.8304 × 10^−5^ cm^2^/s ^d^
Human jejunal permeability	2.7 × 10^−4^ cm/s ^e^
Mean precipitation time	900 s ^f^
Effective particle radius	25 µm ^f^
Drug particle density	1.2 g/mL ^f^
Blood/plasma concentration ratio	0.78 ^g^
Unbound percent in plasma	10% ^g^
First pass extraction	62.89% ^h^
Clearance	0.7 l/h/kg ^i^
Volume of distribution	0.493 l/kg ^i^
Distribution rate constant k_12_	11.40 1/h ^i^
Distribution rate constant k_21_	1.91 1/h ^i^
Elimination half-life	3. 71 h ^d^
**OCCAT Parameters**	
Formulation type	“Buccal patch” ^j^
Drug dose	30 mg ^j^
Contact surface area	2.25 cm^2 k^
Transit model	Normal swallowing ^j^
Saliva production rate	0.6 mL/min ^l^

^a^ literature value is taken from Reference [51]; ^b^ literature value is taken from Reference [52]; ^c^ literature values taken from Reference [24]; ^d^ GastroPlus™ calculated; ^e^ literature value is taken from Reference [53]; ^f^ GastroPlus™ default values; ^g^ literature values taken from Reference [54]; ^h^ literature value is taken from Reference [55]; ^i^ calculated from the in vivo dana [56] using software PKPlus™ module; ^j^ applies for the investigated buccal films; ^k^ corresponds to the film surface area exposed to dissolution medium under the applied dissolution test conditions; ^l^ optimized value [48].

**Table 2:** Temperatures from DSC analysis.

Sample	T_g_, °C	T_m_, °C	T_i_, °C
GA powder	30.41	121.06	n.a.
GB powder	32.35	115.82	n.a.
GA	44.52	85.75	163.33
GB	48.79	87.89	177
GAP	53.11	86.09	147.32
GBP	60.09	94.26	152.55
P		164.33	

**Table 3:** Tensile strength (σ), elongation at break (ε) and elastic modulus (E) of the prepared films.

Sample	E, MPa	σ_m_, MPa	ε, mm/mm
GA	740	19	0.027
GAP	5	1.77	2.87
GB	530	16,21	0.019
GBP	2	0.4	2.85

**Table 4:** Results of the mucoadhesion test, the mucoadhesive force (F_adh_), and the work of adhesion (W_adh_).

Sample	F_adh_, N	St. Dev. N	W_adh_, µJ	St. Dev. µJ
GA	2.09	0.46	1070	150
GAP	2.14	0.44	2350	3390
GB	2.29	0.29	1300	360
GBP	2.32	0.04	910	160

**Table 5:** Analysis of F_adh_.

Factor	F Value	*p* Value
Polymer	0.03063	0.86327
Drug	4.01426	0.06235
Interaction	3.07126	0.09883

**Table 6:** Analysis of W_adh_.

Factor	F Value	*p* Value
Polymer	1.64085	0.21846
Drug	0.88983	0.35955
Interaction	3.14981	0.09496

**Table 7:** Predicted pharmacokinetic parameters following administration of the tested buccal films and the conventional immediate-release (IR) tablets.

Formulation	Dose (mg)	C_max_ (ng/mL)	t_max_ (h)	AUC_0–∞_ (ng h/mL)	F_a_ (%)	F (%)
GAP	30	66.80	2.80	600.99	99.99	99.99
GBP	30	66.80	2.88	612.20	99.99	99.99
IR tablet	80	98.24	1.04	644.23	99.90	35.96

C_max_—maximum plasma concentration; t_max_—time to reach C_max_; AUC—area under the plasma concentration-time curve; F_a_—percent absorbed (entered into the enterocytes/epithelial cells); F—bioavailability.

## Data Availability

Data is contained within the article.
